# Artificial Intelligence Assisted Pharmacophore Design for Philadelphia Chromosome-Positive Leukemia with Gamma-Tocotrienol: A Toxicity Comparison Approach with Asciminib

**DOI:** 10.3390/biomedicines11041041

**Published:** 2023-03-28

**Authors:** Muhammad Naveed, Noor ul Ain, Tariq Aziz, Khushbakht Javed, Muhammad Aqib Shabbir, Metab Alharbi, Abdulrahman Alsahammari, Abdullah F. Alasmari

**Affiliations:** 1 Department of Biotechnology, Faculty of Science & Technology, University of Central Punjab, Lahore 54590, Pakistan; 2 Department of Agriculture, University of Ioannina, 47100 Arta, Greece; 3 Department of Pharmacology and Toxicology, College of Pharmacy, King Saud University, P.O. Box 2455, Riyadh 11451, Saudi Arabia

**Keywords:** Philadelphia chromosome-positive leukemia, chronic myeloid leukemia, artificial intelligence, gamma-tocotrienol, toxicity, asciminib

## Abstract

BCR-ABL1 is a fusion protein as a result of a unique chromosomal translocation (producing the so-called Philadelphia chromosome) that serves as a clinical biomarker primarily for chronic myeloid leukemia (CML); the Philadelphia chromosome also occurs, albeit rather rarely, in other types of leukemia. This fusion protein has proven itself to be a promising therapeutic target. Exploiting the natural vitamin E molecule gamma-tocotrienol as a BCR-ABL1 inhibitor with deep learning artificial intelligence (AI) drug design, this study aims to overcome the present toxicity that embodies the currently provided medications for (Ph+) leukemia, especially asciminib. Gamma-tocotrienol was employed in an AI server for drug design to construct three effective de novo drug compounds for the BCR-ABL1 fusion protein. The AIGT’s (Artificial Intelligence Gamma-Tocotrienol) drug-likeliness analysis among the three led to its nomination as a target possibility. The toxicity assessment research comparing AIGT and asciminib demonstrates that AIGT, in addition to being more effective nonetheless, is also hepatoprotective. While almost all CML patients can achieve remission with tyrosine kinase inhibitors (such as asciminib), they are not cured in the strict sense. Hence it is important to develop new avenues to treat CML. We present in this study new formulations of AIGT. The docking of the AIGT with BCR-ABL1 exhibited a binding affinity of −7.486 kcal/mol, highlighting the AIGT’s feasibility as a pharmaceutical option. Since current medical care only exclusively cures a small number of patients of CML with utter toxicity as a pressing consequence, a new possibility to tackle adverse instances is therefore presented in this study by new formulations of natural compounds of vitamin E, gamma-tocotrienol, thoroughly designed by AI. Even though AI-designed AIGT is effective and adequately safe as computed, in vivo testing is mandatory for the verification of the in vitro results.

## 1. Introduction

Chronic myelogenous leukemia (CML) is caused by a chromosomal translocation t(9;22)(q34;q11.2), which consequences in the BCR-ABL1 chimeric gene as the carcinogenic trigger of (Ph+) leukemia or CML [1]. This fusion gene is a clinical biomarker for CML in addition to a viable treatment approach. In the case of children (CML), it makes up 15% of all instances of myeloid leukemia. Its prevalence rises with age, reaching 1.2 instances per million annually in teenagers [2]. The Philadelphia chromosome (Ph) is what distinguishes CML from other myeloproliferative neoplasms; however, albeit rarely, the Ph+ may also be seen in MPN other than CML [3]. CML can manifest in one of three stages—chronic, accelerated, or blast—and is typically identified in the chronic stage in developed nations.

Asciminib is an allosteric inhibitor that binds to a myristoyl region on the BCR-ABL1 protein. Both natural and altered BCR-ABL1, including the intermediary T315I mutant, are targeted by asciminib [4]. This mechanism of asciminib is different from that of all other ABL kinase inhibitors, as it locks the BCR-ABL1 into an inactive conformation. It exhibits low activity against unmutated BCR-ABL1 and all clinically identified ATP-site mutations, including T315I, though it has significant selectivity for only ABL1 and, presumably, ABL2 kinases. This is due to the unique shape of the myristoyl pocket [5].

Existing ABL inhibitors can be divided into those that target the active conformation of the kinase domain and those that target the inactive kinase domain. These inhibitors compete at the ATP binding sites of these proteins. Since asciminib is distinctive in that it functions as an allosteric inhibitor, attaching to the BCR-ABL1 protein’s myristoyl pocket and immobilizing it in an inactive conformation, it is widely administered for the treatment of (Ph+)leukemia [6]. The health risks of an overdose are likely to coincide with asciminib’s adverse effect profile; therefore, these might include serious hematological abnormalities and/or gastrointestinal side effects, among other concerns.

This study intends to overcome the existing toxicity that prevails in the already administered drugs for (Ph+)leukemia by the utilization of the natural vitamin E compound gamma-tocotrienol as a BCR-ABL1 inhibitor. The artificial intelligence deep learning algorithm application for the de novo drug design of tocotrienol was implemented, and a further toxicity comparison study was performed with asciminib. The AIGT was docked, and furthermore, analysis of AIGT has been proved vital in this study.

## 2. Materials and Methods

### 2.1. 3D Structure Retrieval of Protein

DeepMind developed an artificial intelligence application AlphaFold for the prediction of 3D structures of proteins using the deep learning model [7]. The BCR-ABL1 fusion protein of (CML) was accessed from AlphaFold (https://alphafold.ebi.ac.uk/, accessed on 12 December 2022). AlphaFold is witnessing rapid research, including almost all biological disciplines, and is capable of accurately predicting the 3D models of protein structures with precision.

### 2.2. Binding Sites Identification

A deep neural network-based modulator of protein binding pockets is called DeepSite. A machine learning algorithm that relies on DCNNs for predicting ligand-binding sites in proteins and demonstrates that, given enough training data, consumers can capture binding site characteristic features by providing a comprehensive test set based on more than 7000 proteins from the scPDB database [8]. DeepSite was accessed for free online at (www.playmolecule.org, accessed on 12 December 2022). Through a WebGL graphical interface, the PDB file of the BCR-ABL1 fusion protein was uploaded to the NVIDIA GPU-equipped server for pocket identification and discovery.

### 2.3. Selectivity Search against HCK Gene

The Harvard Program in Therapeutic Sciences (HiTS), in collaboration with the National Institutes of Health (NIH), created the Small Molecule Suite (SMS; https://lsp.connect.hms.harvard.edu/smallmoleculesuite/, accessed on 12 December 2022), a free, open-access platform. It undergoes a technique to evaluate and generate libraries utilizing chemical structure, stage of preclinical studies, user choice, binding selectivity, target coverage and induced cellular phenotypes [9]. The selectivity of molecules against the HCK gene was predicted with this tool. The resultant molecule was further interpreted accordingly.

### 2.4. Post-Refining by MMGBSA Method

Post-refinement of the screening and selection of compounds was done by g_mmgbsa software (https://rashmikumari.github.io/g_mmpbsa/, accessed on 13 December 2022) which is a high-throughput method to validate whether the screening method used earlier is accurate or there are some inactive compounds that could not be detected or screened by the HiTS platform. In this step, docking scores from the Charkasov were also included as a reference [10].

### 2.5. Drug Design by Artificial Intelligence (AI)

The WADDAICA web server is designed to take advantage of both classical and deep learning models for drug design. WADDAICA (https://heisenberg.ucam.edu:5000/, accessed on 13 December 2022) features deep learning models for the scaffold hopping of compounds to alter or generate revolutionary new pharmaceuticals in the first module [11]. The candidate molecule of the vitamin E family, tocotrienol Pubchem id 5282349, was employed as input, and 3 resultant new drug molecules, designed by an AI approach, were regained, respectively. Based on the PDBbind database, the deep learning model implemented in WADDAICA exhibits strong scoring power.

### 2.6. Lipinski’s Rule of 5

Utilizing the Molinspiration tool (https://www.molinspiration.com/, accessed on 14 December 2022) which aids in the prediction of target molecules’ probability of becoming pharmaceutical drugs, the AIGT molecule was investigated [12]. The most important pharmaceutical targets’ bioactivity scores were predicted using this technique, along with important molecular properties (such as logP, polar surface area, the number of hydrogen bond donors and acceptors, and others) were also computed.

### 2.7. Toxicity Screening

ProTox-II (http://tox.charite.de/protox_II, accessed on 16 December 2022) is a virtual lab for the prediction of toxicities of small molecules. For the prediction of various toxicity endpoints, such as acute toxicity, hepatotoxicity, cytotoxicity, carcinogenicity, mutagenicity, immunotoxicity, adverse outcomes pathways (Tox21), and toxicity targets, it combines molecular correlation, pharmacophores, AIGT propensities, and machine-learning models. The predictions are based on findings from both in vivo instances and in vitro assays [13]. The toxicity analysis of asciminib and AIGT was interpreted and compared. The resulting models have demonstrated high performance and have been validated on separate external sets.

### 2.8. ADMET Evaluation

The AIGT and asciminib were employed as input at vnnadmet (https://vnnadmet.bhsai.org/vnnadmet/login.xhtml, accessed on 16 December 2022) for absorption, distribution, metabolism, excretion, and toxicity (ADMET) [14]. Swiftly evaluation of some of the most crucial characteristics of possible drug candidates, such as their potential for producing drug-induced liver damage, microsomal stability, cardiotoxicity, and drug-drug interactions, were figured.

### 2.9. Docking Analysis

The non-covalent docking application DockThor (https://dockthor.lncc.br/v2/, accessed on 17 December 2022), which operates the DockThor-VS web server, uses a pdb file for the ligand and cofactors and a particular input file in pdb for the protein that contains the atom types and partial charges from the MMFF94S49 force field [15]. NGL, a WebGL-based molecular visualization library, creates the visualization of proteins, cofactors, and compounds, as well as the grid position superimposed with the protein. The docking results of AIGT and BCR-ABL1 fusion protein were accessed.

### 2.10. Validation of Docking

The molecular docking algorithm PatchDock, which was developed in 2002 and is based on the shape complementarity theory, was chosen as the docking tool and may be viewed at (https://bioinfo3d.cs.tau.ac.il/PatchDock/, accessed on 20 December 2022). Users simply have to enter specified protein files and ligands in PDB file format in the specified columns with a few optional fields in the docking request form on this freely accessible, highly effective, completely automated online server. The output docking results link displays the top 20 geometry scores, desolvation energies, size of the interface region, and the solution’s actual rigid transformation.

### 2.11. MD Simulations

A platform for comprehending and visualizing three-dimensional biological imaging data is called IMOD (http://imods.chaconlab.org/, accessed on 23 December 2022). This service simulates the representation of complex domain dynamics in macromolecules and discovers potential conformational changes, elastic network possibilities, resolution with a variety of coarse-grained atomic interpretations, and modeling correctness (C Danita et al., 2022). Various image visualization approaches are made available by this software. Models of the image data, which can also be represented as a volume or contour surface, can produce quantitative information.

### 2.12. MMPBSA Analysis

For the prediction of binding free energy and post-refinement process for the screening step, the Molecular Mechanics Poisson Boltzmann Surface Area (MM/PBSA) method was used with the help of g_mmpbsa script (https://rashmikumari.github.io/g_mmpbsa/, accessed on 26 December 2022). In this method, molecular mechanics potential energy, electrostatic forces and van der walls interactions and free energy of solvation, including non-polar and polar interactions, were accessed. From MD simulations, about 80 shots were taken at different intervals to calculate the energy terms. The average of these energy terms was taken to predict the binding free energies. Molecular Mechanics Poisson Boltzmann Surface Area (MM/PBSA) method uses the following equation to calculate or predict the binding free energy between the protein and ligand:

ΔG_binding_ = G_AIGT and BCR-ABL1_ − (G_BCR-ABL1_ + G_AIGT_) (1)

Gx = ⟨EMM⟩ − TS+⟨G_solvation_⟩ (2)

EMM = E_bonded_ + E_non−bonded_ = E_bonded_+ (EvdW + Eelec) (3)

G_solvation_ = G_polar_ + G_non−polar_ (4)

G_non−polar_ = γSASA +b (5)

where G_AIGT and BCR-ABL1_ is the free energy of the whole complex (AIGT and BCR-ABL1), G_BCR-ABL1_ is the free energy of BCR-ABL1 protein, G_AIGT_ is the free energy of ligand AIGT, EMM is the vacuum molecular mechanics potential energy, TS is the entropic contribution into the free binding energy, T is the temperature, S shows the entropy, and G_solvation_ is the free energy of solvation comprised of both polar and non-polar parts. SASA is the solvent-accessible surface area, γ is the coefficient of surface tension, and b is the fitting constant.

## 3. Results

### 3.1. Protein Retrieval

**Figure 1:**
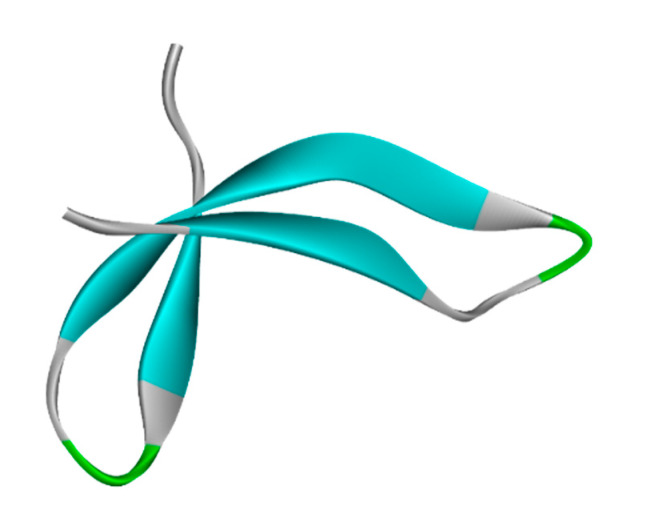
BCR-ABL1 fusion protein 3D structure from AlphaFold.

The BCR-ABL1 fusion protein with Uniport id of A8E194 was assessed from Uniprot. The retrieval of the 3D structure was from Alphafold. The 3D structure has been visualized by Discovery Studio, as shown in Figure 1.

### 3.2. Binding Site Prediction

**Figure 2:**
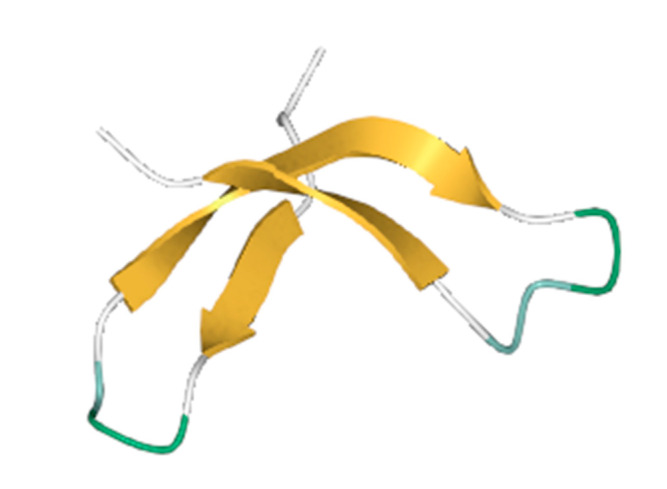
The binding sites of the BCR-ABL1 fusion protein predicted by DeepSite are shown by yellow arrows.

BCR-ABL1 fusion protein binding site predictions were performed with the DeepSite tool. There are three main binding sites predicted, as depicted by yellow arrows in Figure 2. The prediction scores and corresponding exact possible binding positions are displayed in Table 1, respectively.

### 3.3. Selectivity Search against HCK Gene

**Figure 3:**
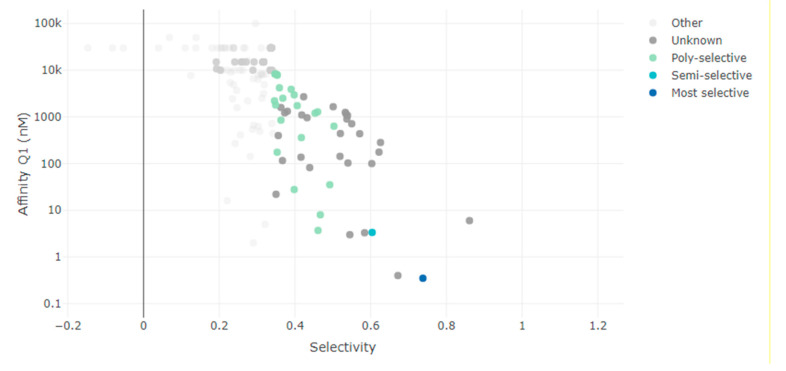
The graph from the small molecular suite showing the most selective, semi-selective and poly-selective molecules that are a hit against the HCK gene. (10k = 10,000, 100k = 100,000).

The target gene for (Ph+) leukemia is HCK. The small molecule suite’s tool selectivity determined the target molecules that are a direct hit for the HCK gene. The graph below is an apparent display of various target molecules with binding affinity up to Q1(nM) on the y-axis and their respective selectivity with the HCK gene on the x-axis. The most selective molecules are shown in dark blue dots, and the other molecules are in grey dots in the graph below (Figure 3). Only one molecule, dasatinib with chEMBL id “CHEMBL1421,” is the most selective molecule targeting the HCK gene.

### 3.4. Post Refinement by MMGBSA Method

The post-refinement results from MMGBSA (Molecular mechanics with generalized born and surface area solvation) validated that there was only one compound that was active while all others were semi-active or inactive compounds, as reported in the upper section. This ultimately showed that no compound was missing or had not been detected by the HiTS database. Table 2 depicts the comparative analysis of the docking scores from the HiTS database and MMGBSA.

### 3.5. Literature Search for Currently Administered Drugs

**Figure 4:**
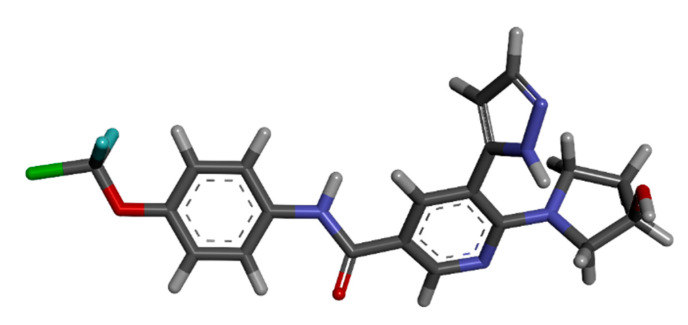
The 3D structure of asciminib from Puchem.

The most recently FDA-approved drug, asciminib, selected as the main target of this study, was studied for its function as an allosteric inhibitor against BCR-ABL1. The physio-chemical properties were looked into, as shown in Table 3, and assessed from Pubchem. The 3D structure of asciminib was retrieved from Pubchem with Puchem id 72165228, as shown in Figure 4 below.

### 3.6. Natural Antineoplastic Agent Search

**Figure 5:**
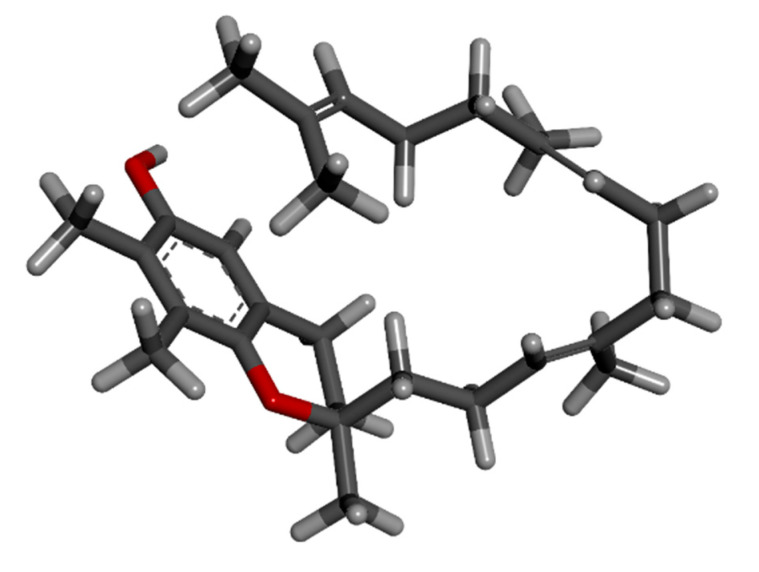
The 3D structure of Gamma-Tocotrienol from Pubchem.

Gamma-tocotrienol is a tocotrienol that has a farnesyl chain at position 2 and methyl groups at positions 2, 7, and 8 of a chroman-6-ol. It is a member of the vitamin E family with powerful anti-cancer capabilities that can fight a variety of malignancies. Gamma-tocotrienol was retrieved from Pubchem with Pubchem id 528249, as shown in Figure 5 below.

### 3.7. Drug Design with Artificial Intelligence

**Figure 6:**
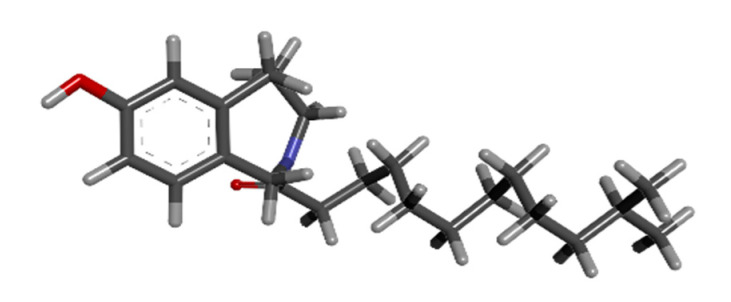
The 3D structure of molecule 1 (AIGT) of Gamma-Tocotrienol AI drug design from WADDAICA.

**Figure 7:**
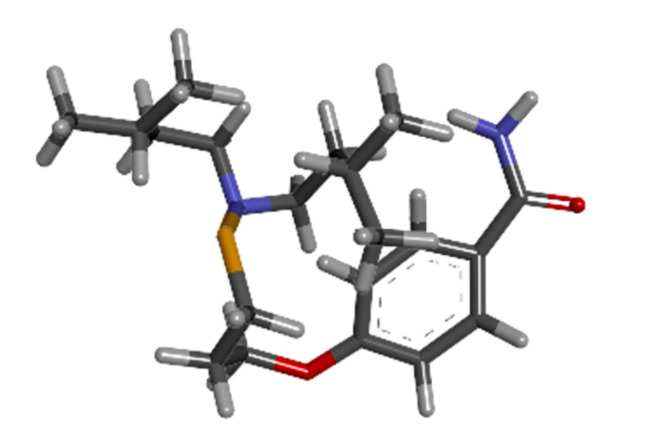
The 3D structure of molecule 2 of Gamma-Tocotrienol AI drug design from WADDAICA.

**Figure 8:**
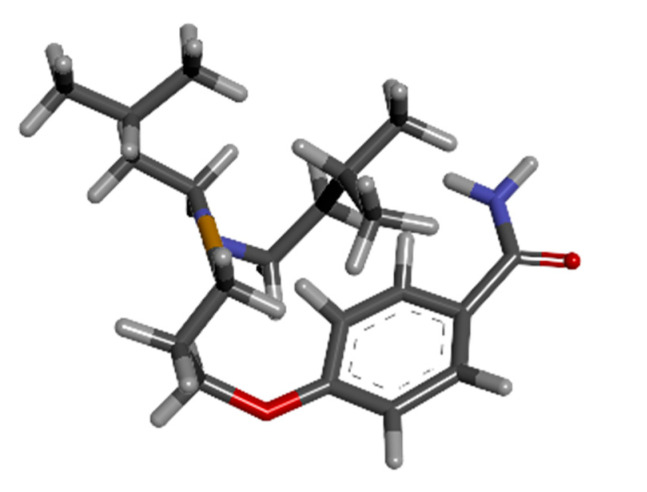
The 3D structure of molecule 3 of Gamma-Tocotrienol AI drug design from WADDAICA.

WADDAICA platform was assessed, and its drug design by AI tool was implemented on gamma-tocotrienol to convert the natural candidate compound into three eligible drug candidates by applying the breakthrough deep learning model. The three molecules designed are shown in Figure 6, Figure 7 and Figure 8 below.

### 3.8. Toxicity Comparison Analysis

The calculation of toxic properties of asciminib is examined, and results depict that there is high predictability against immunotoxicity; moreover, hepatotoxicity and carcinogenicity are also predicted to be active. Moreover, the cytotoxicity and mutagenicity scores are low for being inactive, as shown in Table 4 below. Inactivity against the toxic signaling pathways, as demonstrated in the table, the AIGT has a strong probability of being harmless. Table 5 below demonstrates that there is a high indication that AIGT will not be hepatotoxic, mutagenic, or cytotoxic.

### 3.9. ADMET Comparison Analysis

The late-stage failure of the candidate drug can be prevented by carefully balancing drug-likeness and ADMET (absorption, distribution, metabolism, elimination, and toxicity) during the synthesis of therapeutic molecules, which were analyzed with the help of VNN-ADMET. Table 6 shows that despite being approved by FDA, it is highly predictable that asciminib is again proven to be hepatotoxic. Correspondingly, the AMES test for carcinogenicity is also predicted as positive. Whereas Table 7 depicts that AIGT has no prominent toxicity and thus can prove to be a potential drug candidate.

### 3.10. Lipinski Rule of 5

The results demonstrated in the table below justify that Lipinski’s rule of five is being observed by the AIGT. Molinspiration predicted Lipinki’s rule’s characteristics, such as logP, mass, hydrogen bond donors, hydrogen bond acceptors and molar refractivity. The results of AIGT for Lipinski Rule of 5 are given below in the Table 8.

### 3.11. DockThor Docking Analysis

**Figure 9:**
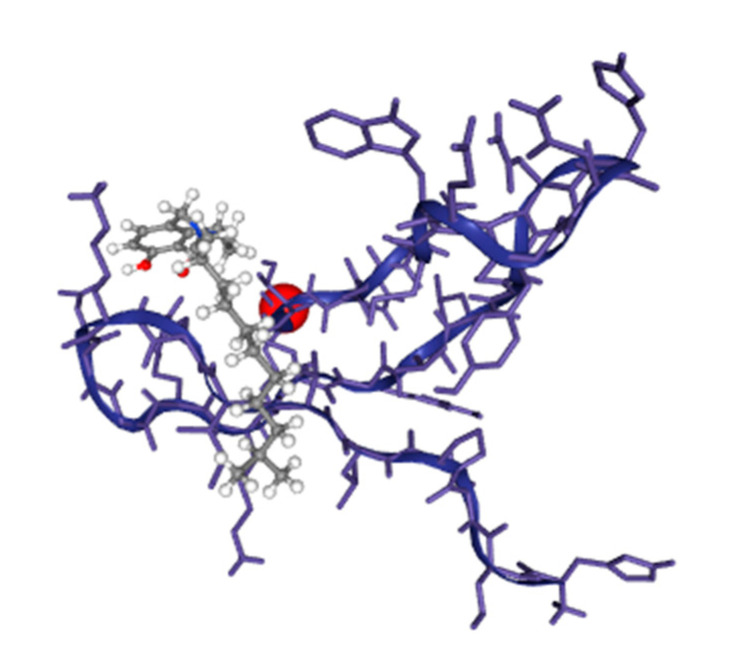
The docked complex of AIGT and BCR-ABL1 where the grey color ligand is the AIGT.

The AIGT and BCR-ABL1 fusion protein were docked with the blind docking option of DockThor. The results depict that the binding affinity is up to −7 kcal/mol, ensuring a valid and high binding energy score. Table 7 below shows the complete results, and the pictorial depiction of docked AIGT and BCR-ABL1 complex is shown in Figure 9. The docking results of AIGT and BCR-ABL1 are given in the Table 9 below.

### 3.12. Docking Results Validation

**Figure 10:**
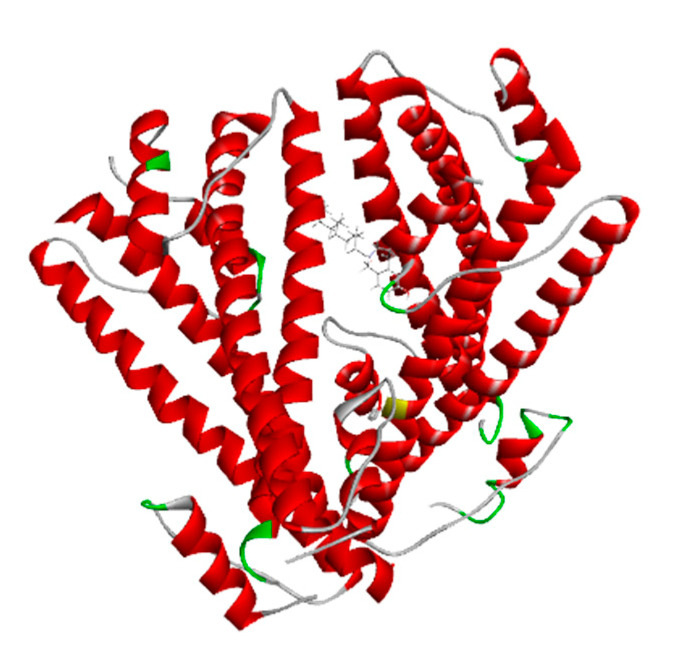
The docked complex of AIGT and BCR-ABL1 from Patchdock.

The docking results of AIGT and BCR-ABL1 obtained from DockThor were further authenticated by means of utilizing the shape complementarity docking server Patchdock. With a score of 5970 and an ACE value of 701.90, model 1 was chosen as the most reliable result. Figure 10 below depicts the docked complex from patchdok.

### 3.13. MD Simulation

**Figure 11:**
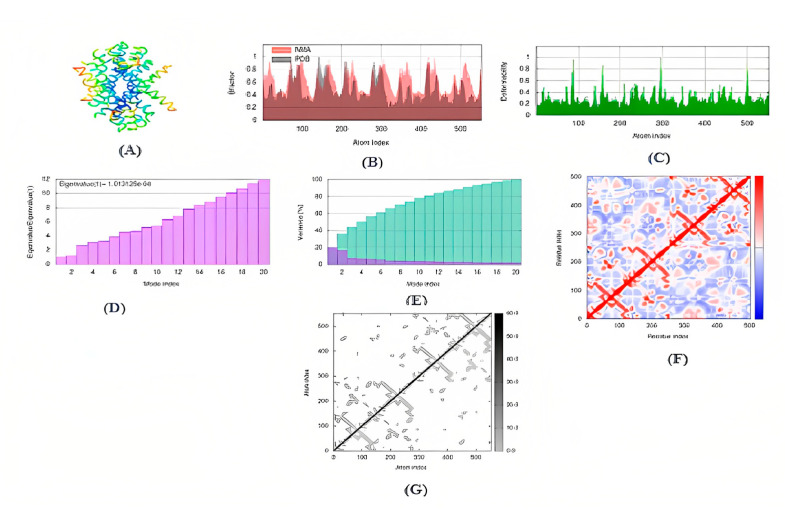
Docked complex of AIGT and BCR-ABL1 molecular dynamic stimulation. (**A**) MNA mobility of 3D structure (**B**) AIGT and BCR-ABL1 docked complex deformability (**C**) B-factor of AIGT and BCR-ABL1 docked complex (**D**) Docked complex AIGT and BCR-ABL1 Eigenvalues (**E**) Docked complex AIGT and BCR-ABL1 variance where individual deviations are shown in purple color, and collective variances are shown in green (**F**) Docked complex AIGT and BCR-ABL1 co-variance map in which the red color shows the correlated area and the blue color depicts anti-correlated motions (**G**) Docked complex AIGT and BCR-ABL1 elastic network where more stiff regions are shown as darker grey in color.

In order to calculate and characterize the docked complex of the AIGT and BCR-ABL1, iMODS considered a number of parameters. The results were explained. The heat map shows that there are several locations that are observed to be directly correlated. A low RMSD value denotes enhanced interactions between the structure’s various residues. The docked complex’s expected Eigon value was 1.013125 × 10^−4^. The results calculated below are shown in Figure 11.

### 3.14. MMPBSA Analysis

Binding free energy and the post-refinement of the final docked complex were predicted by the MM/PBSA method. These calculations showed that the electrostatic force of attraction had dominated the binding energy with a percentage of contribution of 60% in comparison to the Van der Waals interactions with a 21% contribution. So this implies that the electrostatic force has exceeded the force of repulsion and is proven as the main interacting force to be involved in the binding of the protein and the ligand. Thus this analysis validated the binding affinities present in the docked complex. The energy terms, their values and the contributions of percentages are given in Table 10.

## 4. Discussion

Chronic myelogenous leukemia (CML), a slow-growing malignant hematological illness, is a result of 15% of instances of leukemia that are caused [16]. The Philadelphia chromosome, which is formed by a reciprocal translocation that results in a prolonged chromosome 9 and a shorter chromosome 22, is the cause of this illness. The dysregulated BCR-ABL1 fusion carcinogen protein is developed as a result of the translocation, which contributes to the uncontrolled proliferating of white blood cells [17]. Multiple processes involved in cell growth and division, including receptor endocytosis, autophagy, remodeling of the cytoskeleton and actin, cell motility and adhesion, and cell adhesion, depend on ABL1. Additionally, ABL1 translocates into the nucleus, where it assists in apoptosis, the response to DNA damage, and DNA binding activities [4].

The mechanisms of action and toxicity of the medications used to treat CML differ. In a study conducted by Oliver Henke et al. [18]: it was observed that Imatinib’s utilization in the management of CML radically altered the way this disease was addressed and spurred the advent of additional potent targeted protein kinase inhibitors. FDA-approved drugs for initial therapy comprise imatinib as an initial treatment. Furthermore, dasatinib binds to the kinases and prevents them from stimulating growth and is also administered as a treatment. Dasatinib and bosutinib are both regarded as the second line in therapy [19]. Additionally, nilotinib treatment is linked to the transitory increase in serum aminotransferase levels and few incidences of clinically evident acute liver damage [20]. Whereas, although the clinical manifestations of hepatotoxicity are still being precisely defined, occurrences of clinically evident liver problems, progressing hepatic failure, and fatality have been reported in ponatinib clinical studies [21]. Despite hepatoxicity and other health hazardous effects of these drugs, they have been approved by the FDA, and each of these medications is orally ingested to treat (Ph+)leukemia or CML.

Asciminib works as a therapeutic agent by blocking an oncogenic protein that promotes the growth of CML. It functions to inhibit both the wild-type as well as some mutation forms of BCR-ABL1, along with the T315I mutation [17]. Upon its administration, it recognizes and attaches to the myristoyl pocket of the BCR-ABL1 fusion protein, which is distinctive from the ATP-binding domain. An overdose’s side effects are likely to correspond to asciminib’s adverse effect profile; therefore, they may furthermore include severe gastrointestinal problems and hematological abnormalities mainly. Asciminib’s drug-induced death of the proliferating cells was defined by a power model. Additionally, the computational analysis of hepatotoxicity along with other toxicities was computed in this study that validates the administration of asciminib as highly risky. ProTox-II and VNN-Admet simultaneously justify asciminib as crucial for its role in liver damage.

The vitamin E has eight members: four tocopherols, namely α-, β-, δ- and γ-tocopherol, and four tocotrienols in the form of α-, β-, δ- and γ-tocotrienols. Tocotrienols are an underappreciated isomer of vitamin E that has unmatched health advantages [22]. Tocotrienols were rarely used in vitamin E studies until recently, despite their relative superiority to tocopherol and their widespread occurrence in palm oil. This study specifically focuses on the utilization of gamma-tocotrienol since it functions as an apoptosis inducer, a radiation protective agent, a plant metabolite, an antioxidant, an antineoplastic agent, and a hepatoprotective agent [23].

The advanced technique of revolutionary artificial intelligence for drug designing through deep learning algorithms was implemented by assessing the WADDAICA online server. Docking is a valuable tool for screening because it allows for the virtual prediction of how small molecules, such as drugs, will interact with proteins, such as receptors or enzymes. This simulation can provide valuable information about the binding affinity, orientation and energetics of the protein-ligand interaction without the need for experimental techniques. It’s important to note that different scoring functions may have different strengths and weaknesses, and the choice of scoring function depends on the specific problem and the desired trade-off between accuracy and speed. While DockThor Vina may work well for a particular use case, other scoring functions may be better suited for other situations. It’s always a good idea to test and compare multiple scoring functions to determine which is the best fit for a given problem. Post-refining schemes are computational methods that are used to improve the accuracy of protein-ligand docking predictions. Two commonly used post-refining methods are MM/PBSA (Molecular Mechanics/Poisson-Boltzmann Surface Area) and MM/GBSA (Molecular Mechanics/Generalized Born Surface Area). The gamma-tocotrienol was employed for drug design by AI tool for the retrieval of three competent *de novo* drug molecules against the BCR-ABL1 fusion protein. The AIGT was selected as the target candidate based on its drug-likeliness analysis amongst all three. The comparison study of the toxicity of asciminib and AIGT proves that AIGT is not only more efficacious but hepatoprotective as well. Moreover, the ADMET analysis comparison further justifies the findings, The docking of BCR-ABL1-1 with AIGT resulted in the binding affinity of −7.486 kcal/mol hence proving that AIGT is a potential drug candidate. The execution of docking with Patchdock to assure the shape complementarity of the protein and ligand substantially reinforced the docking findings. Thus, the molecular dynamic stimulation also enhanced the reliability of the efficiency of the results.

## 5. Conclusions

A high percentage of patients with Ph+ CML in the chronic phase now experience near-normal life expectancies owing to the availability of BCR-ABL1 tyrosine kinase inhibitors (TKIs). Nonetheless, a significant shortcoming that has been addressed in this study, with asciminib in particular, is the pertinent and escalating problem of toxicity of the currently prescribed drugs. The computational findings indicate that the AIGT is not only non-toxic; however, it is perfectly suited for BCR-ABL1 inhibitors with high binding affinity. The results abide by the Lipinski rule of five; moreover, the substantiated ADMET results are additional arguments in favor of AIGT’s legitimacy as a target medication. Additionally, toxicity comparison between asciminib and AIGT offers further credence for the findings. According to the study, additional experimental evaluations in vivo and in vitro are needed to verify the observations. The research’s outcomes give adequate computational pharmacological knowledge to permit the regulation of a precisely AI-designed AIGT.

## Figures and Tables

**Table 1:** The binding sites center positions of BCR-ABL1 fusion proteins and their scores predicted by DeepSite.

Site No.	Scores	Centers
1	0.80300872	[1.0499999523162842, −0.8100000023841858, 1.7000000476837158]
2	0.798320159	[1.0499999523162842, 1.190000057220459, 1.7000000476837158]
3	0.43639341	[−6.949999809265137, 11.1899995803833, 1.7000000476837158]

**Table 2:** Comparative docking scores of compounds with targeted protein from HiTS database and MMGBSA method.

Compounds	HiTS Docking Score Kcal/Mol	MMGBSA Docking Score Kcal/Mol
TP1589036 or CHEMBL1421	−6.7	−80.78
TJ0975527	−2.5	−78.9
HG08642	−1.7	−67.4
TV54893	−1.5	−64.9
RD5679	−0.6	−54.6
GH0736	−0.5	−52.9
CV6490	−0.2	−34.6

**Table 3:** The physiochemical properties of asciminib retrieved from Pubchem as shown in the Table.

Property Name	Property Value
Molecular Weight	449.8
XLogP3-AA	3
Hydrogen Bond Donor Count	3
Hydrogen Bond Acceptor Count	8
Rotatable Bond Count	6
Exact Mass	449.1066235
Monoisotopic Mass	449.1066235
Topological Polar Surface Area	103 Å^2^
Heavy Atom Count	31

**Table 4:** The toxicity analysis of asciminib from ProTox-II.

Toxicity Model Report of Asciminib				
Classification	Target	Shorthand	Prediction	Probability
Organ toxicity	Hepatotoxicity	Dili	Active	0.50
Toxicity endpoints	Carcinogenicity	Carcino	Active	0.52
Toxicity endpoints	Immunotoxicity	Immune	Active	0.82
Toxicity endpoints	Mutagenicity	Mutagen	Inactive	0.58
Toxicity endpoints	Cytotoxicity	Cyto	Inactive	0.61
Tox21-Nuclear receptor signaling pathways	Aryl hydrocarbon Receptor (AhR)	nr_ahr	Inactive	0.81
Tox21-Nuclear receptor signaling pathways	Androgen Receptor (AR)	nr_ar	Inactive	0.98
Tox21-Nuclear receptor signaling pathways	Androgen Receptor Ligand Binding Domain (AR-LBD)	nr_ar_lbd	Inactive	0.98
Tox21-Nuclear receptor signaling pathways	Aromatase	nr_aromatase	Inactive	0.89
Tox21-Nuclear receptor signaling pathways	Estrogen Receptor Alpha (ER)	nr_er	Inactive	0.85
Tox21-Nuclear receptor signaling pathways	Estrogen Receptor Ligand Binding Domain (ER-LBD)	nr_er_lbd	Inactive	0.94
Tox21-Nuclear receptor signaling pathways	Peroxisome Proliferator-Activated Receptor Gamma (PPAR-Gamma)	nr_ppar_gamma	Inactive	0.92
Tox21-Stress response pathways	Nuclear factor (erythroid-derived 2)-like 2/antioxidant responsive element (nrf2/ARE)	sr_are	Inactive	0.93
Tox21-Stress response pathways	Heat shock factor response element (HSE)	sr_hse	Inactive	0.93
Tox21-Stress response pathways	Mitochondrial Membrane Potential (MMP)	sr_mmp	Inactive	0.67
Tox21-Stress response pathways	Phosphoprotein (Tumor Suppressor) p53	sr_p53	Inactive	0.88
Tox21-Stress response pathways	ATPase family AAA domain-containing protein 5 (ATAD5)	sr_atad5	Inactive	0.92

The green color represents inactive toxicity and red color represents active toxicity in accordance with the probability score ranging from 0–1.0 with higher score showing lesser toxicity.

**Table 5:** The toxicity analysis of AIGT from ProTox-II.

Toxicity Model Report of Drug				
Classification	Target	Shorthand	Prediction	Probability
Organ toxicity	Hepatotoxicity	Dili	Inactive	0.88
Toxicity endpoints	Carcinogenicity	Carcino	Inactive	0.62
Toxicity endpoints	Immunotoxicity	Immune	Inactive	0.98
Toxicity endpoints	Mutagenicity	Mutagen	Inactive	0.70
Toxicity endpoints	Cytotoxicity	cyto	Inactive	0.53
Tox21-Nuclear receptor signaling pathways	Aryl hydrocarbon Receptor (AhR)	nr_ahr	Inactive	0.90
Tox21-Nuclear receptor signaling pathways	Androgen Receptor (AR)	nr_ar	Inactive	0.96
Tox21-Nuclear receptor signaling pathways	Androgen Receptor Ligand Binding Domain (AR-LBD)	nr_ar_lbd	Inactive	0.99
Tox21-Nuclear receptor signaling pathways	Aromatase	nr_aromatase	Inactive	0.93
Tox21-Nuclear receptor signaling pathways	Estrogen Receptor Alpha (ER)	nr_er	Inactive	0.80
Tox21-Nuclear receptor signaling pathways	Estrogen Receptor Ligand Binding Domain (ER-LBD)	nr_er_lbd	Inactive	0.97
Tox21-Nuclear receptor signaling pathways	Peroxisome Proliferator-Activated Receptor Gamma (PPAR-Gamma)	nr_ppar_gamma	Inactive	0.98
Tox21-Stress response pathways	Nuclear factor (erythroid-derived 2)-like 2/antioxidant responsive element (nrf2/ARE)	sr_are	Inactive	0.97
Tox21-Stress response pathways	Heat shock factor response element (HSE)	sr_hse	Inactive	0.97
Tox21-Stress response pathways	Mitochondrial Membrane Potential (MMP)	sr_mmp	Inactive	0.89
Tox21-Stress response pathways	Phosphoprotein (Tumor Suppressor) p53	sr_p53	Inactive	0.93
Tox21-Stress response pathways	ATPase family AAA domain-containing protein 5 (ATAD5)	sr_atad5	Inactive	0.98

The green color represents inactive toxicity and red color represents active toxicity in accordance with the probability score ranging from 0-1.0 with higher score showing lesser toxicity.

**Table 6:** The ADMET analysis results of asciminib show it to be hepatotoxic and negative for the AMES test.

**Query**	**Liver Toxicity**		**Metabolism (Cyp Inhibitors for)**						**Membrane Transporters**			**Others**		
	**DILI**	**Cytotoxicity**	**HLM**	**1A2**	**3A4**	**2D6**	**2C9**	**BBB**	**P-gpInhibitor**	**P-gpSubstrate**	**hERGBlocker**	**MMP**	**AMES**	**MRTD (mg/day)**
	Yes	No	Yes	No	No	No	No	No	Yes	Yes	Yes	No	Yes	207

**Table 7:** The ADMET analysis results of AIGT show it to be non-toxic, especially as an hERG blocker.

**Query**	**Liver Toxicity**		**Metabolism (Cyp Inhibitors for)**						**Membrane Transporters**				**Others**		
	**DILI**	**Cytotoxicity**	**HLM**	**1A2**	**3A4**	**2D6**	**2C1**	**2C19**	**BBB**	**P-gpInhibitor**	**P-gpSubstrate**	**hERGBlocker**	**MMP**	**AMES**	**MRTD (mg/day)**
	Yes	No	Yes	No	No	No	No	No	Yes	Yes	Yes	Yes	No	Yes	25

**Table 8:** The results of AIGT for Lipinski Rule of 5.

Lipinski Rule of 5	logP	Molecular Weight	Hydrogen Bond Donor	Hydrogen Bond Acceptor	Molar Refractivity
Ligand	6.53	381.36 g/mol	1	2	104.7479

**Table 9:** The docking results of AIGT and BCR-ABL1.

Binding Affinity	Total Energy	vdW Energy	Elec. Energy
−7.486	−1.347	−11.844	−15.364

**Table 10:** Prediction of energy terms, values, and contribution percentages calculated by MM/PBSA method.

Terms of Energy	Values(KJ/mol)	Contribution Percentage
Electrostatic force	−15.678	60%
Van der Waals interaction	−11.74	21%
Solvation (polar)	1001.67	0
Solvation (SASA)	−21.78	3%
Binding energy	−7.68	100%

## Data Availability

The data presented in this study are available on request from the corresponding author.
